# Stromal micropapillary component as a novel unfavorable prognostic factor of lung adenocarcinoma

**DOI:** 10.1186/1746-1596-7-3

**Published:** 2012-01-06

**Authors:** Miki Ohe, Tomoyuki Yokose, Yuji Sakuma, Yohei Miyagi, Naoyuki Okamoto, Sachie Osanai, Chikako Hasegawa, Haruhiko Nakayama, Yoichi Kameda, Kouzo Yamada, Takeshi Isobe

**Affiliations:** 1 Department of Pathology, Kanagawa Cancer Center, 1-1-2 Nakao, Asahi-ku, Yokohama, Kanagawa, 241-0815, Japan; 2 Department of Thoracic Oncology, Kanagawa Cancer Center, 1-1-2 Nakao, Asahi-ku, Yokohama, Kanagawa, 241-0815, Japan; 3 Molecular Pathology and Genetics Division, Kanagawa Cancer Center Research Institute, 1-1-2 Nakao, Asahi-ku, Yokohama, Kanagawa, 241-0815, Japan; 4 Cancer Prevention and Cancer Control Division, Kanagawa Cancer Center Research Institute, 1-1-2 Nakao, Asahi-ku, Yokohama, Kanagawa, 241-0815, Japan; 5 Division of Clinical Oncology and Respiratory Medicine, Department of Internal Medicine, Shimane University Faculty of Medicine, 89-1 Enya-cho, Izumo, Shimane, 693-8501, Japan

**Keywords:** lung adenocarcinoma, micropapillary component, stromal micropapillary component, aerogenous micropapillary component, prognostic factor

## Abstract

**Background:**

Pulmonary adenocarcinomas with a micropapillary component having small papillary tufts and lacking a central fibrovascular core are thought to result in poor prognosis. However, the component consists of tumor cells often floating within alveolar spaces (aerogenous micropapillary component [AMPC]) rather than invading fibrotic stroma observed in other organs like breast (stromal invasive micropapillary component [SMPC]). We previously observed cases of lung adenocarcinoma with predominant SMPC that was associated with micropapillary growth of tumors in fibrotic stroma observed in other organs. We evaluated the incidence and clinicopathological characteristics of SMPC in lung adenocarcinoma cases.

**Patients and Methods:**

We investigated the clinicopathological characteristics and prognostic significance of SMPC in lung adenocarcinoma cases by reviewing 559 patients who had undergone surgical resection. We examined the SMPC by performing immunohistochemical analysis with 17 antibodies and by genetic analysis with epidermal growth factor receptor (*EGFR*) and *KRAS *mutations.

**Results:**

SMPC-positive (SMPC(+)) tumors were observed in 19 cases (3.4%). The presence of SMPC was significantly associated with tumor size, advanced-stage disease, lymph node metastasis, pleural invasion, lymphatic invasion, and vascular invasion. Patients with SMPC(+) tumors had significantly poorer outcomes than those with SMPC-negative tumors. Multivariate analysis revealed that SMPC was a significant independent prognostic factor of lung adenocarcinoma, especially for disease-free survival of pathological stage I patients (*p *= 0.035). SMPC showed significantly higher expression of E-cadherin and lower expression of CD44 than the corresponding expression levels shown by AMPC and showed lower surfactant apoprotein A and phospho-c-Met expression level than corresponding expression levels shown by tumor cell components without a micropapillary component. Fourteen cases with SMPC(+) tumors (74%) showed *EGFR *mutations, and none of them showed *KRAS *mutations.

**Conclusions:**

SMPC(+) tumors are rare, but they may be associated with a poor prognosis and have different phenotypic and genotypic characteristics from those of AMPC(+) tumors.

**Virtual Slides:**

The virtual slide(s) for this article can be found here: http://www.diagnosticpathology.diagnomx.eu/vs/9433341526290040.

## Background

A new lung adenocarcinoma classification system has been proposed by the International Association for the Study of Lung Cancer, American Thoracic Society, and European Respiratory Society (IASLC/ATS/ERS) [1]. In this classification, the micropapillary component (MPC) was recommended as a new subtype of lung adenocarcinoma in addition to the lepidic, acinar, papillary, and solid subtypes defined in the 2004 World Health Organization (WHO) classification [2]. MPC was defined as tumor cells growing in papillary tufts lacking fibrovascular cores and may float within alveolar spaces. MPC-predominant lung adenocarcinoma shows a high incidence of nodal metastasis and a poor prognosis [3-8]. MPC-predominant carcinomas developing in various other organs, such as the breast and urinary bladder, known as invasive micropapillary carcinoma, also have a poor prognosis. However, localization of MPC in the lungs is significantly different from that in the other organs; MPC in lung adenocarcinoma is distinguished by floating tumor cells within alveolar spaces (aerogenous micropapillary component, AMPC), while MPC in other organs has been observed primarily in the stroma as invasive components (stromal invasive micropapillary component, SMPC) [3,4].

Few studies have examined lung adenocarcinoma with SMPC [9,10]. Recently, we reported 2 cases of SMPC-predominant lung adenocarcinoma [9]. The proportion of SMPC in both tumors was greater than 50% in area. We observed that SMPC had a strong association with vascular invasion, similar to the cases of SMPC-predominant carcinoma in other organs. However, a large-scale investigation on pulmonary SMPC has not been conducted.

The aims of this study included: (1) clarifying the incidence of SMPC in lung adenocarcinoma; (2) elucidating the clinicopathological characteristics of the tumor; and (3) determining the prognoses of the SMPC-positive (SMPC(+)) tumors and comparing them with those of SMPC-negative (SMPC(-)) tumors. We reviewed 559 resected lung adenocarcinomas for this study with performing immunohistochemical and genetic analysis.

## Methods

### Patients

We analyzed 565 consecutive cases of primary lung adenocarcinoma treated by surgical resection at the Kanagawa Cancer Center between February 2007 and December 2010. Formalin fixation of the resected lung tissue was performed within 48 hours to reduce the loss of immunohistochemical antigen expression and degeneration of DNA. Six patients who had received preoperative chemotherapy were excluded. A total of 559 cases were enrolled in the study. The median follow-up time was 634.5 days (range, 28-1512 days). All patients provided informed consent, and the studies were performed according to the requirements of the institutional review board of Kanagawa Cancer Center.

### Pathological review

Excised specimens were fixed in a solution of 10% buffered formaldehyde, and the sections were embedded in paraffin. Next, 4-μm-thick sections, including the largest cut surface of the tumor, were prepared and stained using hematoxylin and eosin (HE) as well as alcian blue and elastica-van-Gieson (AB-EVG) to detect cytoplasmic mucin production and the elastic fiber framework. Lymphatic invasion and pulmonary metastasis were evaluated on HE sections. Vascular and pleural invasion was evaluated in AB-EVG sections. Sections were reviewed by 2 observers (M.O. and T.Y.) who were unaware of the clinical data. Tumor size was measured as the maximal diameter on the cut sections of the lung. Pathological stage was determined based on the criteria of the 7^th ^TNM classification of Union of International Cancer Control [11].

### Histological definition of micropapillary components

**Figure 1:**
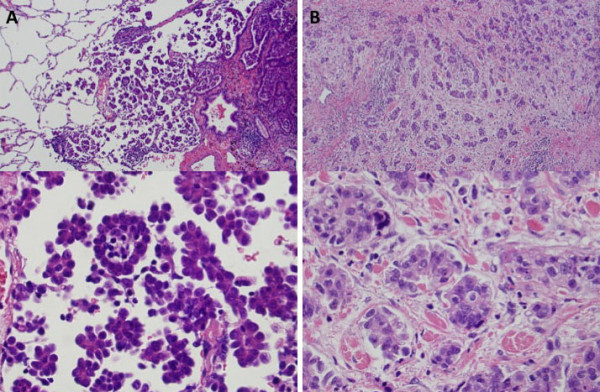
**Microscopic features of micropapillary component in the lung adenocarcinoma stained with hematoxylin-eosin (HE)**. A, AMPC. AMPC is the micropapillary component in which tumor cells are floating within alveolar spaces. B, SMPC. SMPC are tumor cells observed in the stroma and consisting of a papillary component with a tuft lacking central fibrovascular cores surrounded by lacunar spaces. (A, B: upper panel: magnification, ×100; lower panel: magnification, ×400) SMPC, stromal micropapillary component; AMPC, aerogenous micropapillary component.

Histopathological diagnosis of lung adenocarcinoma was determined according to the IASLC/ATS/ERS international multidisciplinary classification of lung adenocarcinoma [1]. Comprehensive histological subtyping was performed on the primary tumor and divided by percentage into 5 distinctive subtypes: lepidic, acinar, papillary, micropapillary, and solid, totaling 100% per tumor. We defined the subtype as positive when it occupied at least 1% of the entire tumor. We classified a micropapillary subtype into 2 components, AMPC and SMPC, using the following criteria: AMPC is widely recognized in the lungs as tumor cells floating within alveolar spaces, and SMPC includes papillary components consisting of tufts lacking central fibrovascular cores, surrounded by lacunar spaces and identified as invasive components in the stroma as previously described [9] (Figure 1A and 1B). Additionally, a tumor area without micropapillary components was defined as a non-micropapillary component (nMPC).

### Tumor tissue microarray (TMA) synthesis

TMAs were constructed using a manual tissue-arraying instrument (KIN-4; Azumaya, Tokyo, Japan) as previously described [12], and specimens were punched using a stylet 3 mm in diameter.

### Immunohistochemistry

The 17 antibodies used for immunohistochemical characterization of tumor cells in TMA in this study are listed in Table 1. Immunohistochemical staining was performed as follows. TMA recipient blocks were cut into 4-μm-thick sections and mounted on silane-coated slides. HE staining was performed on initial sections to verify histology. The remaining sections were deparaffinized in xylene and dehydrated in a graded alcohol series, and endogenous peroxidase was blocked using 3% hydrogen peroxide in absolute methyl alcohol. Heat-induced epitope retrieval was performed for 20 min at 95°C in 0.02 mol/L citrate buffer (pH 6.0) in samples fixed with 10% formalin if necessary. The slides were rinsed using deionized water and incubated with primary antibodies. They were then washed 3 times in phosphate-buffered saline and incubated with EnVision+ System-HRP (DAKO, Glostrup, Denmark). The reaction products were visualized using 3-3'-diaminobenzidine tetrahydrochloride, and sections were counterstained using hematoxylin. Additionally, a similar staining method was used for anti-podoplanin antibody (clone D2-40, pre-diluted; Ventana, Tucson, AZ, USA) to evaluate lymphatic permeation.

**Table 1:** Antibodies

Classification/Antibody	Clone	Dilution	Source
**Cellular adhesion molecules**			
E-cadherin	NCH-38	1:100	DakoCytomation, Carpinteria, CA, USA
CD44	DF1485	1:400	Novocastra, Newcastle upon Tyne, UK
Laminin5γ2	4G1	1:50	DakoCytomation, Glostrup, Denmark
Growth factor			
VEGF-C	Polyclonal	1:50	Abcam, Cambridge, UK
Apoptosis-associated proteins			
bcl2	124	1:50	DakoCytomation, Glostrup, Denmark
p53	DO-7	Pre-diluted	Nichirei, Tokyo, Japan
cleaved caspase-3	Polyclonal	1:400	Cell signaling, Danvers, MA, USA
Mucin-related proteins			
MUC1	Ma695	1:100	Novocastra, Newcastle upon Tyne, UK
MUC6	CLH5	1:100	Novocastra, Newcastle upon Tyne, UK
Hypoxia induced protein			
HIF-1α	EP1215Y	1:500	Abcam, Cambridge, UK
Others			
TTF-1	8G7G3/1	1:100	DakoCytomation, Carpinteria, CA, USA
SP-A	PE10	1:100	Dako, Kyoto, Japan
Vimentin	V9	Pre-diluted	DakoCytomation, Carpinteria, CA, USA
Ki-67	MIB-1	1:50	Dako, Glostrup, Denmark
LYVE1	15A5B2	1:400	Oriental Yeast, Tokyo, Japan
c-Met	EP1454Y	1:200	Abcam, Cambridge, UK
Phospho-c-Met	Polyclonal	1:800	Stressgen, Ann Arbor, MI, USA

VEGF-C, vascular endothelial growth factor-C; HIF-1α, hypoxia induced factor 1-α; TTF-1, thyroid transcription factor-1; SP-A, surfactant apoprotein A; LYVE1, lymphatic vessel endothelial hyaluronan receptor 1.

### Calculation of staining scores

Immunostaining was scored based on staining intensity and percentage of positively stained cells, with 2 observers evaluating immunostained samples independently. When the observers gave different scores to immunostained samples, the slides were reviewed together under a multiheaded microscope until a consensus was reached. Sections were classified by staining intensity as negative (total absence of staining), 1+ (weak staining), 2+ (moderate staining), or 3+ (strong staining). Staining scores were calculated by multiplying the percentage of positive tumor cells per section (0-100%) by the staining intensity; scores obtained ranged from 0 to 300. Expression of p53, cleaved caspase-3, and Ki-67 were determined by counting 300 tumor cells under a high power field (×400) and results are shown as the percentage of positive cells.

### Mutation analysis

Mutation analyses of *EGFR *gene exons 19 and 21 and *KRAS *gene codons 12 and 13 were performed using loop-hybrid mobility shift assays and gene sequencing procedures described elsewhere [13].

### Statistical analysis

All calculations were performed using SPSS software (Dr. SPSS II for Windows Standard version 11.0; SPSS Inc., Chicago, IL, USA). The Chi-square for independence or Fisher's exact probability test was performed to analyze differences in patient characteristics between the 2 groups. The Fisher's exact probability test was performed if there were 5 or fewer observations in a group. For univariate analysis, all cumulative survival was estimated using the Kaplan-Meier method, and differences in variables were calculated using the log-rank test. Multivariate regression analysis was conducted according to the Cox proportional hazard model. The Mann-Whitney *U *test was used to compare staining scores. Differences were considered significant when the *P *value was less than 0.05.

## Results

### Clinicopathological characteristics of patients with SMPC

**Figure 2:**
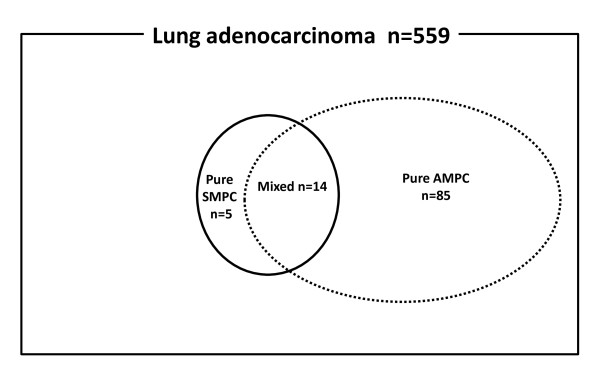
**Venn diagram of patients included in the present study**. Among the 559 cases of lung adenocarcinoma, 104 cases had MPC. Nineteen cases had SMPC (SMPC(+) tumors, the area enclosed by continuous line), and 99 had AMPC (AMPC(+) tumors, the area enclosed by dotted line). A mixture of SMPC and AMPC was observed in 14 patients, SMPC without AMPC in 5 and AMPC without SMPC in 85. MPC, micropapillary component; SMPC, stromal micropapillary component; AMPC, aerogenous micropapillary component.

Figure 2 shows a Venn diagram of the relationship between the micropapillary component sets in the 559 patients examined in this study. SMPC was observed in 19 patients (3.4%) and AMPC in 99 (17.7%) patients. A mixture of SMPC and AMPC was observed in 14 patients, pure SMPC without AMPC in 5 patients, and pure AMPC without SMPC in 85 patients. A micropapillary pattern was observed in 50-100% in 2 SMPC tumor and less than 50% in 17 SMPC tumors. No SMPC(+) tumors were completely replaced by SMPC. Clinicopathological characteristics of patients with SMPC(+) and SMPC(-) tumors are summarized in Table 2. Patients with SMPC(+) tumors were significantly found to be at a more advanced stage, larger than 30 mm in diameter, and have more frequent lymph node metastasis compared to those with SMPC(-) tumors. Pleural, lymphatic, and vascular invasion were observed more often in patients with SMPC(+) tumors than in those with SMPC(-) tumors. (68% vs. 17%, *P *< 0.001; 74% vs. 15%, *P *< 0.001; 74% vs. 22%, *P *< 0.001, respectively). No significant differences in age, gender, or smoking status were observed between patients with SMPC(+) and SMPC(-) tumors.

**Table 2:** Clinicopathological characteristics of patients with SMPC

	all	%	SMPC				*P *value
				
			(-)	%	(+)	%	
No.	559		540	97	19	3	
Age							
Median	67		67		67		0.219*
Range	23-87		23-87		40-76		
Gender							
Female	288	52	282	52	6	32	0.077**
Male	271	48	258	48	13	68	
Smoking status							
Nonsmoker	284	51	276	51	8	42	0.596**
Smoker	275	49	264	49	11	58	
BI Average	369		364		502		
Tumor size							
Average(mm)	25		25		35		
Range(mm)	5-140		5-140		15-75		
< 30 mm	396	71	388	72	9	47	< 0.001*
≥ 30 mm	163	29	152	28	10	53	
Pathological stage							
IA	363	65	360	67	4	21	< 0.001**
IB	95	17	88	16	6	32	
IIA	36	6	31	6	5	26	
IIB	13	2	13	2	0	0	
IIIA	42	8	39	7	3	16	
≥ IIIB	10	2	9	2	1	5	
Lymph node metastasis							
NX	69	12	68	13	1	5	
N0	420	75	409	75	11	58	0.002**
≥ N1	70	13	63	12	7	21	
Pleural invasion							
Negative	452	80	446	83	6	32	< 0.001**
Positive	107	20	94	17	13	68	
Lymphatic invasion							
Negative	466	83	461	85	5	26	< 0.001**
Positive	93	17	79	15	14	74	
Vascular invasion							
Negative	427	76	422	78	5	26	< 0.001**
Positive	132	24	118	22	14	74	

* Mann-Whitney's U test

** Chi-square for independence test

No., number of patients; BI, Brinkman index = (number of cigarettes per day) × (duration of years); SMPC, stromal micropapillary component; AMPC, aerogeneous micropapillary component

### Survival analysis

**Figure 3:**
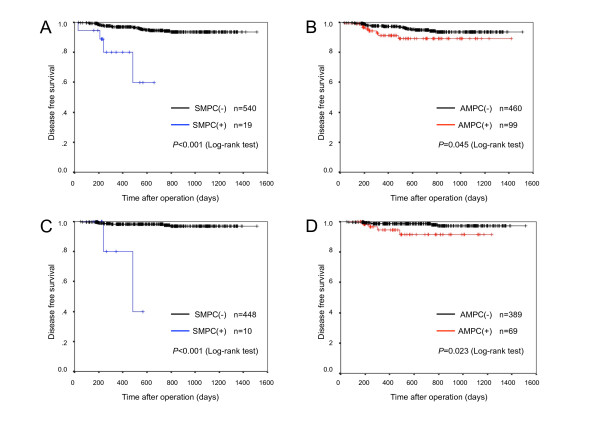
**Cumulative disease-free survival rates of patients according to presence of SMPC and AMPC**. A, B are cumulative disease-free survival rates in all stage, and C, D are that of in p-stage I. Cumulative disease-free survival rates stratified by presence of SMPC are shown in A and C, and those stratified by presence of AMPC are shown in B and D. In all stage and in p-stage I, SMPC(+) tumors and AMPC(+) tumors had significantly poorer outcomes. Outcomes of SMPC(+) tumors were more significantly negative than those of AMPC(+) tumors. SMPC, stromal micropapillary component; AMPC, aerogenous micropapillary component.

Among all stage patients, median follow-up time was 654 days (range, 33-1512 days) in SMPC(-) tumors, 240 days (range, 28-661 days) in SMPC(+) tumors, 664 days (range, 28-1512 days) in AMPC(-) tumors, and 467 days (range, 36-1412 days) in AMPC(+) tumors. Among the stage I patients, median follow-up time was 767 days (range, 59-1343 days) in SMPC(-) tumors, 192 days (range, 227-485 days) in SMPC(+) tumors, 767 days (range, 59-1343 days) in AMPC(-) tumors, and 836 days (range, 140-1233 days) in AMPC(+) tumors. Recurrence occurred in 28 of 559 cases. SMPC(+) tumors recurred in 4 of 19 in all stage and in 2 of 10 in p-stage I, and AMPC(+) tumors recurred in 8 of 99 cases and 4 of 69 cases, respectively. In all stage, disease-free survival (DFS) of patients with SMPC(+) tumors was significantly poorer than that in patients with SMPC(-) tumors (Figure 3A, P < 0.001); the same result was observed in patients with AMPC(+) and AMPC(-) tumors (Figure 3B, P = 0.045,). In p-stage I patients, DFS of those with SMPC(+) tumors showed significantly poorer outcome than that of patients with SMPC(-) tumors (Figure 3C, P < 0.001); the same result was observed between patients with AMPC(+) and AMPC(-) tumors (Figure 3D, P = 0.023).

In univariate analysis, high pathological stage (*P *< 0.001), pleural invasion (*P *< 0.001), lymphatic invasion (*P *< 0.001), vascular invasion (*P *< 0.001), SMPC(+) (*P *< 0.001), and AMPC(+) tumors (*P *= 0.045) showed an unfavorable influence on survival for all stage, and pleural invasion (*P *< 0.001), lymphatic invasion (*P *< 0.001), vascular invasion (*P *< 0.001), SMPC(+) (*P *< 0.001), and AMPC(+) tumors (*P *= 0.023) showed an unfavorable influence on survival for p-stage I (Table 3, 4). In multivariate analysis, pathological stage (*P *= 0.028), lymphatic invasion (*P *= 0.009), and vascular invasion (*P *= 0.011) were identified as significant independent prognostic factors for all stage (Table 3). Though not observed for all stage, the presence of SMPC(+) tumors (*P *= 0.035) was identified as a significant independent prognostic factor for p-stage I, as well as lymphatic invasion (*P *= 0.020) and vascular invasion (*P *= 0.049) (Table 4). The presence of AMPC(+) tumors was not a significant prognostic factor for all stage or p-stage I.

**Table 3:** Impact of potential prognostic factors on DFS of patients of lung adenocarcinoma in all stage by univariate and multivariate analysis

	**No**.	%	Univariate analysis	Multivariate analysis		
				
			*P *value	Hazard ratio	95% CI	*P *value
Total	559					
Age						
< 65	213	38	0.388	1.000		
≥ 65	346	62		1.933	0.849-4.402	0.116
Gender						
Female	288	52	0.768	1.000		
Male	271	48		0.807	0.232-2.803	0.735
Smoking status						
Non-smoker	284	49	0.560	1.000		
Smoker	275	51		1.164	0.342-3.956	0.808
Tumor size						
< 30 mm	396	71	0.059	1.000		
≥ 30 mm	163	29		0.819	0.338-1.985	0.658
Pathological stage						
I	458	82	<0.001	1.000		
II, III, IV	101	18		2.768	1.113-6.884	0.028
Pleural invasion						
Negative	452	81	<0.001	1.000		
Positive	107	19		0.848	0.345-2.083	0.719
Lymphatic invasion						
Negative	466	83	<0.001	1.000		
Positive	93	17		3.430	1.363-8.634	0.009
Vascular invasion						
Negative	427	76	<0.001	1.000		
Positive	132	24		3.309	1.312-8.350	0.011
SMPC						
Negative	540	97	<0.001	1.000		
Positive	19	3		1.871	0.528-6.630	0.332
AMPC						
Negative	460	83	0.045	1.000		
Positive	99	17		1.132	0.450-2.845	0.792

DFS, disease free survival; No., number of patients; SMPC, stromal micropapillary component; AMPC, aerogeneous micropapillary component; CI, confidence interval.

**Table 4:** Impact of potential prognostic factors on DFS of patients of lung adenocarcinoma in p-stage I by univariate and multivariate analysis

	**No**.	%	Univariate Analysis	Multivariate analysis		
				
			*P *value	Hazard ratio	95% CI	*P *value
Total	458					
Age						
< 65	172	38	0.394	1.000		
≥ 65	286	62		2.191	0.474-10.131	0.316
Gender						
Female	249	54	0.063	1.000		
Male	209	46		0.157	0.014-1.787	0.136
Smoking status						
Non-smoker	248	54	0.204	1.000		
Smoker	210	46		0.768	0.117-5.052	0.784
Tumor size						
< 30 mm	358	78	0.264	1.000		
≥ 30 mm	100	22		0.304	0.037-2.504	0.268
Pleural invasion						
Negative	402	88	< 0.001	1.000		
Positive	56	12		1.519	0.328-7.040	0.593
Lymphatic invasion						
Negative	415	91	< 0.001	1.000		
Positive	43	9		5.016	1.295-19.434	0.020
Vascular invasion						
Negative	390	85	< 0.001	1.000		
Positive	68	15		4.494	1.006-20.081	0.049
SMPC						
Negative	448	98	< 0.001	1.000		
Positive	10	2		9.028	1.164-70.031	0.035
AMPC						
Negative	389	98	0.023	1.000		
Positive	69	2		1.825	0.378-8.808	0.454

DFS, disease free survival; No., number of patients; SMPC, stromal micropapillary component; AMPC, aerogeneous micropapillary component; CI, confidence interval.

### Immunohistochemical findings

We evaluated immunohistochemical profiles of SMPC, AMPC, and nMPC. These lesions were evaluated in TMAs for 33 cases, including 19 SMPC(+) tumors and 14 pure AMPC tumors. The latter 14 tumors were selected from 85 pure AMPC tumors according to operation date, patient age, gender, and smoking status to match clinical background factors between SMPC and AMPC. nMPC was generally included in TMA cores of SMPC and AMPC. The total number of TMA was 19 SMPC and 28 AMPC. Staining scores are summarized in Table 5.

**Table 5:** Staining Scores in SMPC, AMPC and nMPC lesions

Classification/Antibody	SMPC	AMPC	nMPC
Cellular adhesion molecules			
E-cadherin	215.3*	143.9	187.1
CD44	60.8^‡^	205.9^¶^	141.3
Laminin5γ2	69.4	36.9	60.3
Growth factor			
VEGF-C	294.4	296.4	282.1
Apoptosis-associated proteins			
bcl2	13.2	11.1	21.8
p53^§^	36.4	26.1	45.0
cleaved caspase-3^§^	0.3	0.2	0.4
Mucin-related proteins			
MUC1	169.7	182.5	202.1
MUC6	0.0	0.0	0.0
Hypoxia induced protein			
HIF-1α	1.8	2.4	2.9
Others			
TTF-1	267.9	289.3	248.6
SP-A	45.2^‡^	82.6	123.2
Vimentin	112.1	117.9	72.1
Ki-67^§^	22.6	16.9	16.2
LYVE1	98.9	107.2	101.9
c-Met	217.2	253.8	211.4
Phospho-c-Met	34.2^‡^	50.0	88.0

SMPC, stromal micropapillary component; AMPC, aerogeneous micropapillary component; nMPC, non-micropapillary component; VEGF-C, vascular endothelial growth factor-C; HIF-1α, hypoxia induced factor 1-α; TTF-1, thyroid transcription factor-1; SP-A, surfactant apoprotein A; LYVE1, lymphatic vessel endothelial hyaluronan receptor 1.

§ Positivity rate of positive tumor cells in 300 tumor cells (percentage).

* The difference in staining scores between SMPC and AMPC is statistically significant (*P *= 0.020).

‡ The differences in staining scores between SMPC and nMPC are statistically significant for CD44 (*P *= 0.011), and SP-A (*P *= 0.024) and phospho-c-Met (*P *= 0.011) expression.

¶ The difference in staining scores between AMPC and nMPC is statistically significant (*P *= 0.015).

**Figure 4:**
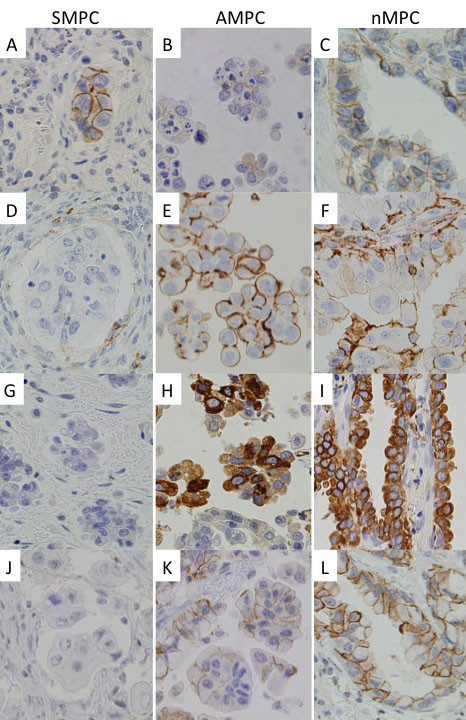
**Photomicrographs of immunohistochemistry. E-cadherin (A-C); CD44 (D-F); SP-A (G-I); Phospho-c-Met (J-L)**. Compared with AMPC, increased E-cadherin and decreased CD44 membrane immunostaining were found in SMPC. Moreover, SP-A cytoplasm and Phospho-c-Met membrane immunostaining were decreased in SMPC (×400). SMPC, stromal micropapillary component, left panels; AMPC, aerogenous micropapillary component, middle panels; nMPC, non-micropapillary component, right panels.

In cellular adhesion molecules, E-cadherin staining scores in patients with SMPC, AMPC, and nMPC were 215.3, 143.9, and 187.1, respectively, and although the differences were not significant between patients with SMPC or nMPC and between patients with AMPC or nMPC (*P *= 0.312, 0.127, respectively), staining scores of SMPC were significantly higher than those for patients with AMPC (*P *= 0.020) (Figure 4A-C). CD44 staining scores in SMPC, AMPC, and nMPC were 60.8, 205.9, and 141.3, respectively. The CD44 expression level in SMPC was significantly lower than in AMPC (*P *< 0.001) and significantly higher than that in nMPC lesions (*P *= 0.015) (Figure 4D-F).

For other antibodies, staining scores of surfactant apoprotein A (SP-A) in the SMPC, AMPC, and nMPC were 45.2, 82.6, and 123.2, respectively, and although the difference was not significant between AMPC and nMPC (*P *= 0.203), the staining score in SMPC was significantly lower than those in nMPC (*P *= 0.024) (Figure 4G-I). Similarly, staining scores of phospho-c-Met in SMPC, AMPC, and nMPC were 34.2, 50.5, 88.0, respectively, and staining scores in SMPC were significantly lower than those in nMPC (Figure 4J-L).

### Mutation analysis

Mutation analysis was performed in 33 patients for whom TMAs were constructed for immunohistochemical analysis. Table 6 summarizes the results of the mutation analysis. Although no cases examined possessed the *KRAS *mutations, *EGFR *mutations were detected in 20 cases (61%): 14 in patients with SMPC(+) tumors (74%) and 6 in patients with SMPC(-) tumors (43%). There was no significant association between the existence of SMPC and *EGFR *mutations. Among the 20 cases with *EGFR *mutations, 7 had deletions at exon 19, 13 had a point mutation at exon 21, and there were no multiple mutations. Among the 13 cases with a point mutation at exon 21, 12 had an L858R mutation and one had an L861Q mutation.

**Table 6:** Mutation analysis

	total	%	SMPC(+) cases	%	SMPC(-) cases	%	*P *value
No.	33		19		14		
*EGFR *mutation							
Negative	13	39	5	26	8	57	0.076*
Positive	20	61	14	74	6	43	
ex19	7	35^§^	5	36^§^	2	33^§^	0.664*
ex21	13	65^§^	9	64^§^	4	67^§^	
*KRAS *mutation							
Negative	33	100	19	100	14	100	-
Positive	0	0	0		0		

No., number of patients; EGFR, epidermal growth factor receptor; SMPC, stromal micropapillary component

* Fisher's exact probability test

§ Rate of positive cases at ex19 and 21 in *EGFR *mutation positive cases, respectively.

## Discussion

The present study revealed the incidence of SMPC(+) lung adenocarcinoma in consecutive surgical cases to be 3.4%, which is lower than that of AMPC(+) lung adenocarcinoma (17.7%). In non-pulmonary organs, the incidence of invasive micropapillary carcinoma was reported to be 7% in breast carcinoma [14], 0.9% in urinary bladder cancer [15], and 9.4% in colon cancer [16]. Generally, invasive micropapillary carcinomas occur infrequently in any organ.

Prognosis of lung adenocarcinoma with MPC has been reported to be worse and have the potential for high malignancy [17,18], but no studies have separately evaluated SMPC and AMPC. We showed that SMPC(+) tumors as well as AMPC(+) tumors are associated with several biological factors including tumor size, lymph node metastasis, advanced stage disease, and pleural and lymphovascular invasion. Univariate analysis also revealed the presence of SMPC and AMPC as a significant predictor of unfavorable outcome. However, the most remarkable finding was observed in multivariate analysis: among the patients in p-stage I, patients with not AMPC but SMPC showed a significantly poorer DFS than those without MPC. We used immunohistochemistry with monoclonal antibody D2-40 against lymphatic endothelium in TMA specimens and found that lymphatic vessels are involved within SMPC areas in 4 (21%) of 19 SMPC(+) tumors (data not shown). When compared with AMPC(+) tumors, SMPC(+) tumors significantly more often showed pleural, lymphatic, and vascular invasion than AMPC(+) tumors (68% vs. 33%, *P *= 0.004; 74% vs. 30%, *P *< 0.001; 74% vs. 41%, *P *= 0.010, respectively). Therefore, these data suggest that a strong association between SMPC(+) tumors and pleural and lymphovascular invasion may in part explain their aggressive behavior.

Moreover, we investigated the immunohistochemical differences between SMPC and AMPC. In the study, we observed high E-cadherin expression and low CD44 expression in SMPC. Phospho-c-Met expression generally decreases in SMPC to a greater extent than in AMPC. Recently, it has been suggested that E-cadherin repression and CD44 expression are associated with the epithelial-mesenchymal transition (EMT), which was thought to lead to tumor invasion [19,20]. Additionally, Elliot et al. reported that hepatocyte growth factor (HGF) and c-Met signaling promotes EMT in breast cancer [21], and Orian-Rousseau et al. reported that CD44 is strictly required for c-Met activation by HGF in human carcinoma [22]. Consistent with these data, EMT may not occur in SMPC despite its existence in the stroma, or invasion of SMPC may occur through a different invasion mechanism from EMT. Our immunohistochemical findings of SMPC showed lower expression of SP-A than that of nMPC. Many studies have reported that SP-A deletion is correlated with patient survival, and reduced SP-A in MPC may be an excellent indicator for poor prognosis in small-size lung adenocarcinoma [23,24]. Reduced SP-A may contribute to an unfavorable outcome of SMPC(+) tumors.

Some studies have reported a significant association between the presence of MPC and *EGFR *mutations and effectiveness of EGFR tyrosine kinase inhibitor (EGFR-TKI) for MPC(+) tumors [25-28]. Since SMPC of lung adenocarcinoma may be associated with a high incidence of *EGFR *mutations, EGFR-TKI may be effective against SMPC(+) tumors. Patients with these pathological features of lung adenocarcinoma may benefit from EGFR-TKI as postoperative chemotherapy or first-line chemotherapy of relapsed lung adenocarcinoma.

In conclusion, we observed SMPC(+) adenocarcinoma. The incidence of SMPC(+) tumors is low, and SMPC(+) tumors have a different prognostic impact compared to AMPC(+) tumors. Particularly for the early stage tumors, SMPC(+) tumors have different pathobiological characteristics from AMPC(+) tumors, and SMPC(+) tumors frequently contain the *EGFR *mutation. Therefore, it is important to determine the presence of SMPC in lung adenocarcinoma, particularly p-stage I tumors, and the presence of SMPC should be noted in a pathology report to alert the clinician to the possibility of poor prognosis.

## List of abbreviations

AMPC: aerogenous micropapillary component; SMPC: stromal micropapillary component; MPC: micropapillary component; TMA: tumor tissue microarray; DFS: disease-free survival.

## Competing interests

The authors declare that they have no competing interests.

## Authors' contributions

MO and TY designed the study, performed clinical and pathological investigation, and wrote the drafts. YS and YM participated in pathological and genetical investigation. NO participated in statistical investigation. SO performed the histological and immunohistochemical evaluation. CH assisted the clinical investigation. HN participated in managing and operating the patients. YK assisted the pathological investigation. KY participated in collecting clinical data and images. TI participated in its design and coordination and helped to draft the manuscript. All authors read and approved the final manuscript.
